# Assessment of the Heavy Metal Contamination of Roadside Soils Alongside Buddha Nullah, Ludhiana, (Punjab) India

**DOI:** 10.3390/ijerph19031596

**Published:** 2022-01-30

**Authors:** Jaskaran Kaur, Sartaj Ahmad Bhat, Navdeep Singh, Sandip Singh Bhatti, Varinder Kaur, Jatinder Kaur Katnoria

**Affiliations:** 1 Department of Botanical and Environmental Sciences, Guru Nanak Dev University, Amritsar 143005, India; jaskaranbot.rsh@gndu.ac.in (J.K.); singh.sandip87@gmail.com (S.S.B.); 2 River Basin Research Center, Gifu University, 1-1 Yanagido, Gifu 501-1193, Japan; sartajbhat88@gmail.com; 3 Waste Reprocessing Division, CSIR-National Environmental Engineering Research Institute (CSIR-NEERI), Nehru Marg, Nagpur 440020, India; 4 School of Chemical Engineering and Physical Sciences, Lovely Professional University, Phagwara 144001, India; navdeep.24963@lpu.co.in; 5 Centre for Advanced Studies, Department of Chemistry, Guru Nanak Dev University, Amritsar 143005, India; varinder_texchem@gndu.ac.in

**Keywords:** Buddha Nullah, heavy metals, roadside soil, pollution indices, Pearson correlation, multivariate analysis

## Abstract

The present study was carried out to determine the physico-chemical characteristics and heavy metal contents in roadside soil samples collected during 2 sampling periods (September 2018 and April 2019) from 8 different roadside sites lying parallel to the Buddha Nullah, an old rivulet, flowing through Ludhiana, (Punjab) India. The contents (mg/kg) of seven metals (cadmium, chromium, cobalt, copper, lead, nickel and zinc) were estimated using a flame atomic absorption spectrophotometer. Among the metals analyzed, the contents of Cd, Co, Cu, Pb and Zn were found above the permissible limits. The results of the index of geoaccumulation (Igeo), contamination factor (CF), contamination degree (Cdeg), modified contamination degree (mCdeg), the Nemerow pollution index (PI) and pollution load index (PLI) indicate a moderate to high heavy metal contamination of the analyzed soil samples. The results of the potential ecological risk factor (ERi) and potential ecological risk index (RI) indicate a low to moderate risk of heavy metals in the studied soil samples. The Pearson correlation analysis revealed that most of the variables exhibited a statistically significant correlation with one or more variables during the two samplings. Multivariate analysis demonstrates that contents of heavy metals in the study area are influenced by anthropogenic and geogenic factors.

## 1. Introduction

Soil, all over the world in the past years, has been contaminated very rapidly due to different anthropogenic activities, such as effluent discharges from domestic and industrial sources, crumbs of vehicular parts, mining activities, power stations and metallurgical industries [1]. Various types of contaminants, such as heavy metals, pesticides and polyaromatic hydrocarbons, have been documented to enter the soil ecosystem through direct and indirect human activities [2,3]. Among the various contaminants, heavy metals have been recognized as potential carcinogens that fall under the category of most hazardous pollutants due to their direct toxicity, ecological risks and non-degradable nature [4]. Apart from these, heavy metals upon exposure via inhalation, ingestion or dermal contact can pose both carcinogenic as well as non-carcinogenic effects on human beings [5,6,7]. The ultimate threat of heavy metals in the soil is due to their persistent nature and their potential to become bio-accumulated in food crop plants [8,9]. Once these heavy metals enter food crops, they can pose adverse effects upon the consumption of contaminated vegetables and grains. Soil pollution, on account of heavy metals, has turned out to be a serious problem in developing countries due to the increasing number of pollution sources [10]. Various anthropogenic activities, including automobile emissions, traffic activities and industrial activities, can cause heavy metals to diffuse into urbanized environments [11]. Based on the above, roadside soil, street dust, and plants can be exposed to significant levels of metals, owing to both vehicle emissions and carried harmful chemicals [12,13,14]. The burning of fossil fuels, vehicle wear (tires, body and brakes) and vehicular fluids all contribute to increased metal levels in the environment [15]. It has been observed that roadside soil is highly contaminated with various heavy metals, namely Ni, Cd, Zn, Cu and Pb [12,13,16]. Many studies found that human activities are the primary source of metal contamination in different environmental samples, such as soil, dust, sediments and plants. Thus, the study of heavy metal pollution in soils is the need of the hour. Many studies have been conducted to explore the spatial distribution of heavy metal pollution in roadside soils [17,18,19,20,21,22,23,24].

The bioaccumulation of different metals in crop plants depends on the physico-chemical characteristics of the soil. The parameters, such as pH, electrical conductivity, availability of various cations and anions, play a key role in metal availability to the plants from the soil. The physico-chemical characteristics of soil differ from place to place and from time to time, depending on the parent material, due to integrated effects of natural factors, for example, climate conditions and anthropogenic activities, such as emission from industrial, domestic and vehicular sources [25]. It is well established that soil physico-chemical characterization plays a key role in exploring the composition of soil and evaluating soil pollution [26,27]. Many studies across the world have been conducted to explore the physico-chemical characteristics of roadside soil in different regions [10,28,29,30,31,32].

Soil, being the major sink for the accumulation of different contaminants, such as heavy metals released through anthropogenic practices, needs immediate attention [33]. Hence, it has become imperative to comprehend levels the soil pollution in different areas all over the world. In past decades, the general criteria adopted for the evaluation of soil pollution mainly focused on the physico-chemical characterization of soil. However, a number of parameters, the huge data and the variability of data, can designate the level of pollution, but make it difficult to compare the pollution levels of various sites. To overcome this problem, a broad ranging approach has been applied by various researchers to assess the soil pollution, which includes the usage of various indices, such as the heavy metal pollution index (HPI), geoaccumulation index (Igeo), enrichment factor (EF), contamination factor (CF) and ecological risk index (RI) [21,24,34,35,36,37,38,39,40,41].

Keeping this in view, the present work is designed to investigate the heavy metal contents and physico-chemical characteristics in roadside soils alongside the Buddha Nullah, Ludhiana. Furthermore, the level of contamination and ecological risk of heavy metals is also measured using various pollution indices, viz., the geoaccumulation index (Igeo), contamination factor (CF), degree of contamination (Cdeg), modified degree of contamination (mCdeg), the Numerow pollution index (PI), pollution load index (PLI), potential ecological risk factor (ER_i_) and the potential ecological risk index (RI).

## 2. Material Methods

### 2.1. Study Area and Sample Collection

The present study was carried out during September 2018 (Sampling 1) and April 2019 (Sampling 2) along the midstream region of Buddha Nullah, Ludhiana (Punjab), India. Ludhiana is the most polluted and populous city of Punjab State (India) and Buddha Nullah, a seasonal water stream, passes through this industrial city. Buddha Nullah receives domestic waste water along with the partially treated or untreated industrial effluent from industrial units related to electroplating, cycle manufacturing, machine parts, hosiery and dyeing loaded with mainly toxic and heavy metals. This deteriorates the quality of water and soil in the vicinity of Buddha Nullah.

**Figure 1:**
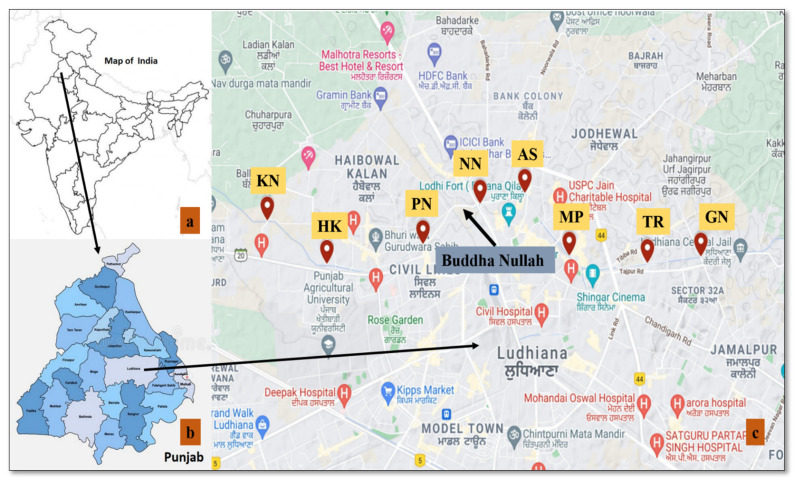
Distribution of the sampling sites and location of the study area (Buddha Nullah, Ludhiana (Punjab), India. (Image source: (**a**) scanned outline map of India (https://oppidanlibrary.com/map-of-india/outline-map-of-india-2/ (accessed on 20 December 2021)); (**b**) map of Punjab (https://www.shutterstock.com/image-vector/punjab-map-political-administrative-districts-name-1877078080 (accessed on 20 December 2021)) and (**c**) Google Earth (with tagged sampling sites) and modifications). Sample codes as mentioned in Table 1.

Hence, the area around Buddha Nullah in the Ludhiana city areas was selected for the present study. The study area lies between a 30°55′08.5″ N and 30°55′31.2″ N latitude, and a 75°53′56.3″ E and 75°47′31.5″ E longitude (Figure 1).

To investigate the soil quality, soil samples were collected from the 8 different roadside sites lying parallel to the Buddha Nullah (Table 1). For the collection of soil samples, the soil was dug to the depth of 20–25 cm. Soil samples were taken from 4–5 locations and pooled to constitute the sample of the particular site. Soil samples were stored in clean and airtight polyethylene bags and brought to the laboratory for further analysis. Soil samples were then air-dried in the laboratory for 3 days at room temperature. The soil was physically cleaned, homogenized and sieved through a size 2 mm sieve for further analysis, removing visible remnants of leaves and other waste elements.

### 2.2. Sample Preparation and Analysis

For the analysis of the physico-chemical parameters, the soil extract (1: 5 *w*/*v*) was prepared. A total of 20 g of the collected soil sample was added in 100 mL of distilled water. This solution was maintained at room temperature in a mechanical shaker for 12 h before being filtered through Whatman No. 1 filter paper. The filtrate was called soil extract and was used to determine the different physico-chemical parameters, viz., pH, alkalinity, electrical conductivity (EC), calcium, sodium, magnesium and potassium. The pH and electrical conductivity of the soil samples were measured using a pH meter (Model: µ pH system 361; make: Systronics). The sodium and potassium content of the soil samples were determined using a Flame Photometer (Model-128; make: Systronics). The dry combustion method was used to determine the total organic carbon in the soil [42]. For the bulk density (BD) estimation, a core measuring cylinder (100 mL) was utilized [43]. The sieve and sedimentation method was used to determine the soil texture [44]. Different sizes of the soil particles are classified as follows: sand, 0.5–2.00 mm; silt, 0.002–0.5 mm and clay, 0.002 mm. The alkalinity, calcium and magnesium were measured using the titrimetric method [45].

For the heavy metal estimation, the soil samples were digested using aqua regia following the method described by Ehi-Eromosele et al. [46] with minor modifications. For this purpose, 1 g of finely ground soil sample was digested slowly with 2–3 mL of aqua regia (1:3, *v*/*v*: HNO_3_:HCl) on a hot plate in a fume hood. After evaporation, 2–3 mL of HCl was added to the mixture and again evaporated until white fumes appeared, indicating the complete digestion of soil sample. The digested soil samples were filtered through Whatman No.1 filter paper and diluted to a final amount of 50 mL with double distilled water. This filtrate was used further for the estimation of heavy metal contents, viz., Cd, Cr, Co, Cu, Pb, Ni and Zn, using atomic absorption spectrophotometer (Model: 240 FS of Agilent). For the Cd, Cr, Co, Cu, Pb, Ni and Zn estimations, cathode lamps were set at wavelengths of 228.80 nm, 357.90 nm, 240.70 nm, 324.70 nm, 217.00 nm, 232.0 nm and 213.90 nm, respectively. The airflow rate was set at 13.50 L min^−1^ for all the heavy metals, while the acetylene flow rate was kept at 2.00 L min^−1^ for Cd, Co, Cu, Pb, Ni and Zn, but 2.90 L min^−1^ for Cr. Lamp currents were set at 4.00 mA, 7.00 mA, 7.00 mA, 4.00 mA, 5.00 mA, 4.00 mA and 5.00 mA for Cd, Cr, Co, Cu, Pb, Ni and Zn, respectively. Chemicals and reagents of analytical grade were employed throughout the experiment. For the preparation and dilution of the reagents, standards and samples, only double distilled water was utilized. For each metal, calibration curves were carefully produced using standards prepared by the dilution of stock standards (10,000 mg/L in 5% HNO_3_) obtained from Agilent Technologies, Pvt. Limited, Bengaluru, Karnataka, India. Furthermore, blanks were run on a frequent basis to confirm the quality of the analysis, and washings with double distilled water were administered at regular intervals to avoid analyte deposition in the instrument. Triplicate analysis, calibration of the instruments with analytical grade metal standards, reference standard checks and reagent blank checks were among the laboratory quality assurance and quality control approaches used in the assessment.

### 2.3. Pollution and Ecological Risk Assessment

For the exploration of the pollution level caused by the heavy metals in the studied area, various indices were calculated, such as an index of geoaccumulation (Igeo), contamination factor (CF), contamination degree (Cdeg), modified contamination degree (mCdeg), Nemerow’s pollution index (PI), pollution load index (PLI), potential ecological risk factor (ER_i_) and potential ecological risk index (RI). Table 2 presents a brief overview of these soil contamination indices.

### 2.4. Statistical Analysis

The analysis of all the experiments was performed in triplicates and the data were presented as the mean ± standard error. The correlation between different soil quality parameters was calculated by applying the Pearson correlation method using SPSS 16.0 software. The multivariate statistical methods, viz., factor analysis/principal component analysis and cluster analysis using SPSS 16.0 software, were used for the interpretation of the soil quality monitoring data.

## 3. Results and Discussions

The results of the various physico-chemical characteristics analyzed for the soil samples collected from roadside sites lying along the Buddha Nullah, Ludhiana, during Sampling 1 (September 2018) and Sampling 2 (April 2019) are shown in Table 3.

The pH measures the hydrogen ion concentration and is considered as an important parameter that indicates the acidic or alkaline nature of the soil. The the pH of soil samples was found to be slightly acidic to slightly alkaline and remained within the prescribed limits of 6.5–8.5 provided by Ramachandra et al. [54]. The pH was observed as minimum (6.66) for the Geeta Nagar (GN) sample collected during Sampling 1, and maximum (7.78) for the Madhopuri (MP) sample, which was collected during Sampling 2. In the present study, the content (mS/cm) of EC ranged from 0.14 to 2.60 and 0.16 to 2.16 during the first and second sampling, respectively. The EC of all the soil samples was found to be less than the salt concentration limit for the non-saline soil extract, i.e., 4.5 mS/cm, indicated that the soils under examination were not saline in nature [55]. Our findings are backed up by Celenk and Kiziloglu [58], who found that the roadway soil in Sakarya city, Turkey, is “salt free or non-saline”, which could be attributed to a lack of various ions in the soil mixture. A side variation in EC of roadside soils was also reported in earlier studies [59,60,61]. The bulk density (BD) values for roadside soil samples collected during sampling 1 and 2 ranged from 0.97 g/cc (Tajpur road: TR) to 1.28 g/cc (Arvindra street: AS) and from 0.90 g/cc (TR) to 1.23 g/cc (AS), respectively.

Soil texture is one of the important parameters, which is defined as the stable aggregates formed by the arrangement of soil particles of varying sizes, such as sand, silt and clay. The content of clay dominated in all roadside soil samples studied during the two samplings (Table 3). The overall content (%) of sand, silt and clay particles in the roadside soil samples ranged as 1.66 (HK: Haibowal Kalan)–29.58 (KN: Kitchlu Nagar), 8.58 (MP: Madho Puri)–60.43 (TR: Tajpur Road) and 36.07 (TR: Tajpur Road)–84.59 (Haibowal Kalan, HK), respectively. The clay particles hold cations over their surface due to being negatively charged. Therefore, these soils are chemically most active due to the fact that absorbed fraction offers an incessant source of cations to the soil and the roots of the plants. In the present work, the alkalinity of all the roadside soil samples ranged from 500 mg/kg to 5000 mg/kg. The content (mg/kg) of calcium in the the roadside soil samples varied from 120.24 (MP: Malakpur) to 3139.60 (HK: Haibowal Kalan). All soil samples showed magnesium content below the safe limits of 0–500 mg/kg presented by the Indian Standard Institution [56], Awashthi [57] and Alghobar and Suresha [62], except at the Haibowal Kalan (HK) site during Sampling 1, i.e., 609.12 mg/kg. The high content of magnesium and sodium in roadside soil is linked to vehicle movement, coal combustion, road pavement materials and deicing substance use [63]. During the present work, the minimum concentration of potassium was observed as 249.83 mg/kg and 219.03 mg/kg for the RP site, while the maximum was 2800.83 mg/kg and 2637.22 mg/kg for the GR site, during the first and second sampling, respectively. Degraded conditions may be to blame for the drop in potassium content in the roadside soil. Basumatary and Bordoloi [64] and Boruah and Nath [65] discovered that a layer of organic matter boosts potassium retention in soils considerably. Furthermore, a degraded environment accelerates the leaching of minerals (such as K^+^) and may reduce the amount of accessible potassium in the soil. This could explain why potassium levels are lower at some roadside and higher in all other roadside sites. According to Ghiri et al. [66], the distribution of different potassium forms in soils differed significantly. This discrepancy could be due to the changes in soil chemical characteristics, as well as the extent to which potassium salts leached in distinct soil series. Soil organic matter (SOM) is an important parameter of the soil that affects nutrient retention and heavy metals in the soil, as well as helps in the growth of plants [67]. The values of SOM ranged from 1.33 to 10.73% in the roadside soil samples.

The results of a metal analysis of the roadside soil samples are presented in Table 4. Wilson et al. [68] documented that soil physico-chemical properties have a strong influence on the environmental fate and the transport of heavy metals concentrations in soils. The presence of some metals, such as chromium, copper and cobalt, in the soil are very essential for the metabolic activities of plants, whereas cadmium and lead are known plant toxins and human carcinogens [69,70]. It was found that the cadmium content in most of the roadside soil samples (0.03 to 0.46 mg/kg) of the study area were above the safe limit of 0.06 mg/kg [71]. The main reason for the high cadmium content in the study area is attributed to various anthropogenic actions, i.e., waste disposal from different industrial units, along with roadside sites in the study area [72,73]. The concentration (mg/kg) of chromium in the roadside soil samples in the study area varied from 9.32–104.62 and was less than the safe limit of 100 mg/kg [71], except at one site, i.e., Geeta Nagar (GN: 104.62 mg/kg) during Sampling 1. It was observed that most of the soil samples showed cobalt content (4.15 mg/kg to 11.43 mg/kg) above the typical concentration of 8.00 mg/kg [71]. The concentration (mg/kg) of Cu, Pb, Ni and Zn in the soil samples varied from 10.05–198.29, 8.83–69.17, 9.18–182.88 and 53.88–303.58. It was found that copper contents in all the roadside soil samples during Sampling 2 were above the typical soil concentration, i.e., 20 mg/kg presented by Agarwal [71]. Lead content in most of the roadside soil samples was observed to be greater than the soil limit, i.e., 10 mg/kg [71], except at one site, i.e., Haibowal Kalan (HK: 8.83 mg/kg) during Sampling 2. The major anthropogenic sources in the roadside soils accountable for the higher lead content were attributed to be a settlement of coal fly ash released from industries in the vicinity of the study area and vehicular emissions [72,74]. The content of zinc in all the roadside soil samples during both samplings, 1 and 2, were observed to be higher than the typical concentration of soil, i.e., 50 mg/kg [71]. Zinc pollution in roadside soils was caused by traffic-related activities, such as vehicular emissions and the weathering of crash barriers [67,75,76]. Similarly, variations in the contents of heavy metals in the roadside soils were also reported by various authors around the world [77,78,79,80,81,82].

### 3.1. Pollution and Ecological Risk Assessment

The level of contamination of the roadside soil samples with studied heavy metals (Cd, Cr, Co, Cu, Pb, Ni and Zn) were determined by using various indices, such as an index of geoaccumulation (Igeo), contamination factor (CF), contamination degree (Cdeg), modified contamination degree (mCdeg), the Nemerow pollution index (PI), potential ecological risk factor (ER_i_), potential ecological risk index (RI) and pollution load index (PLI).

The range of index of geoaccumulation (Igeo) for seven heavy metals, viz., Cd, Cr, Co, Cu, Pb, Ni and Zn, was −2.56 to 1.64, −2.49 to 0.99, −1.85 to −0.39, −1.90 to 2.40, −1.76 to 1.21, −0.66 to 2.61 and −0.30 to 0.45, respectively, for the roadside soil samples, indicating that the level of contaminants varied from no contamination to moderate contamination of the soil samples from the study area (Table 5). The moderate contamination of roadside soils with Cu metal was also documented by several authors [85,86,87]. Various early studies also reported the variations from no contamination to moderate contamination with Co in different roadside soils [88,89].

**Figure 2:**
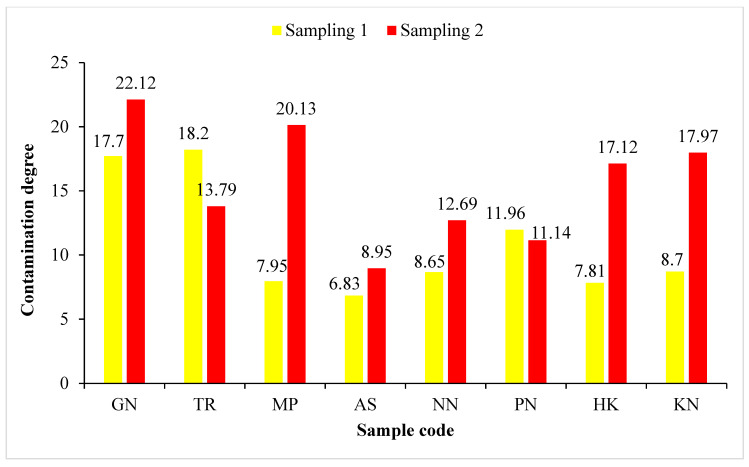
Contamination degree (Cdeg) of roadside soil samples collected from the vicinity of Buddha Nullah stream, Ludhiana (Punjab), India, during Sampling 1 (September 2018) and Sampling 2 (April 2019). Sample codes as mentioned in Table 1.

**Figure 3:**
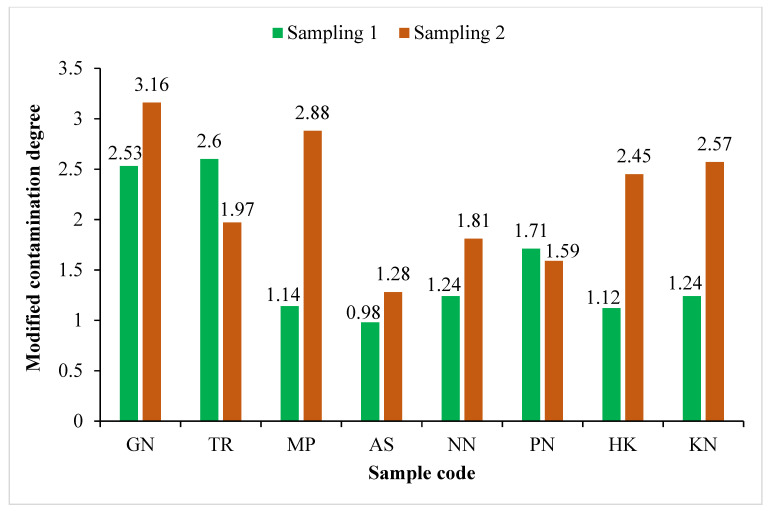
Modified contamination degree (mCdeg) of roadside soil samples collected from the vicinity of Buddha Nullah stream, Ludhiana (Punjab), India, during Sampling 1 (September 2018) and Sampling 2 (April 2019). Sample codes as mentioned in Table 1.

**Figure 4:**
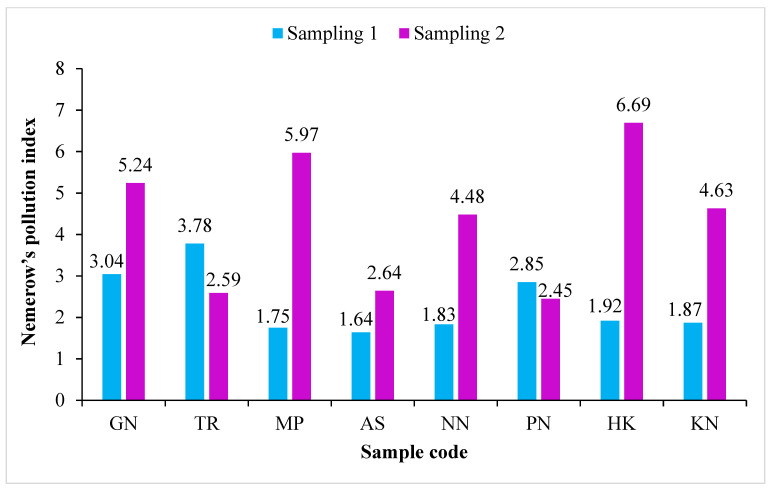
The Nemerow pollution index (PI) of the roadside soil samples collected from the vicinity of Buddha Nullah stream, Ludhiana (Punjab), India, during Sampling 1 (S1: September 2018) and Sampling 2 (April 2019). Sample codes as mentioned in Table 1.

The pollution level, depending on the values of contamination factor (CF), can be classified as low contamination (<1), moderate contamination (1–3), considerable contamination (3–6) and very high contamination (>6). On the basis of the results obtained from CF, the roadside soils were found to be low contamination to moderate contamination with Cr (0.27 to 2.99) and Co (0.42 to 1.14), low contamination to considerable contamination with Cd (0.26 to 4.68), Pb (0.44 to 3.46) and Zn (0.76 to 4.28), whereas they were low contamination to heavy contamination with Cu (0.40 to 7.93) and Ni (0.46 to 9.14), as shown in Table 6. Contamination degree (Cdeg), in the present study, indicated that the pollution of the roadside soil (7.81 to 22.12) samples was low degree to moderate degree of contamination (Figure 2). The results of the modified contamination degree (mCdeg) indicated that the quality of the soil in the study area fell into the category of low (0.98) to moderate (3.16) degree of contamination (Figure 3)**.** According to Nemerow [90], the soil quality can be categorized as unpolluted (<0.7), slightly polluted (0.7–1), moderately polluted (1–2), severely polluted (2–3) and heavily polluted (>3) using the Nemerow pollution index (PI). The PI of the roadside soils was found to be in the range of 1.64 to 6.69, indicating moderate to heavy pollution with studied heavy metals (Cd, Cr, Co, Cu, Pb, Ni and Zn), as shown in Figure 4.

**Figure 5:**
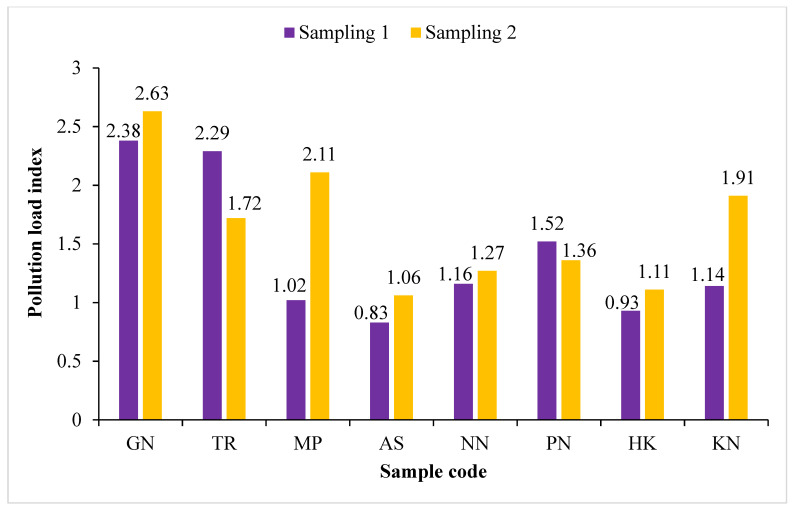
Pollution load index (PLI) of roadside soil samples collected from the vicinity of Buddha Nullah stream, Ludhiana (Punjab), India, during Sampling 1 (September 2018) and Sampling 2 (April 2019). Sample codes as mentioned in Table 1.

The results of ER_i_ show no to low risk with Cr (0.53 to 5.98), Co (2.08 to 5.72), Cu (2.01 to 39.66), Pb (2.21 to 14.31), Ni (2.29 to 45.72) and Zn (0.76 to 4.28), while low to considerable risk with Cd (7.65 to 140.31) in the roadside soil samples from the study area (Table 7). The potential ecological risk index (RI) demonstrates low (43.02) to moderate (189.58) risk in the soil samples (Table 7). According to Tomlinson et al. [53], the pollution load index (PLI) can be divided into three classes: unpolluted (PLI < 1), baseline levels of pollutants (PLI = 1) and polluted (PLI > 1). The result of the PLI was found to be in the range of 0.83 to 2.63, which indicates that soils were unpolluted to polluted (Figure 5). The results of PLI in the present study are in corroboration with Charzynski et al. [91], where it was demonstrated that the PLI values were in the range of 0.1 to 2.8 in their studies of soils in northwest Poland.

### 3.2. Statistical Analysis

The Pearson correlation analysis was carried out among the soil quality parameters to measure the relationship between the parameters. The correlation between the physico-chemical parameters and heavy metals was analyzed during 2 sampling periods and the significance of value was checked at the level of *p* < 0.05 and *p* < 0.01 (Table 8 and Table 9). Electrical conductivity showed a statistically significant positive correlation with calcium and magnesium during Sampling 1, which indicated that a rise in the levels of these nutrients leads to a subsequent increase in EC. A statistically significant negative correlation was observed between the soil organic matter (SOM) and bulk density (BD) during both samplings. Kizilkaya and Dengiz [92] stated that the loss of organic matter resulted in a higher bulk density in a study. In the case of the heavy metals, a statistically significant positive correlation was observed between Cd-Cu, Cd-Pb, Cr-Ni, Cr-Zn, Cu-Pb, Cu-Zn, Pb-Zn, Ni-Zn and Cr-Pb, Ni-Zn, during Sampling 1 and 2, respectively.

**Figure 6:**
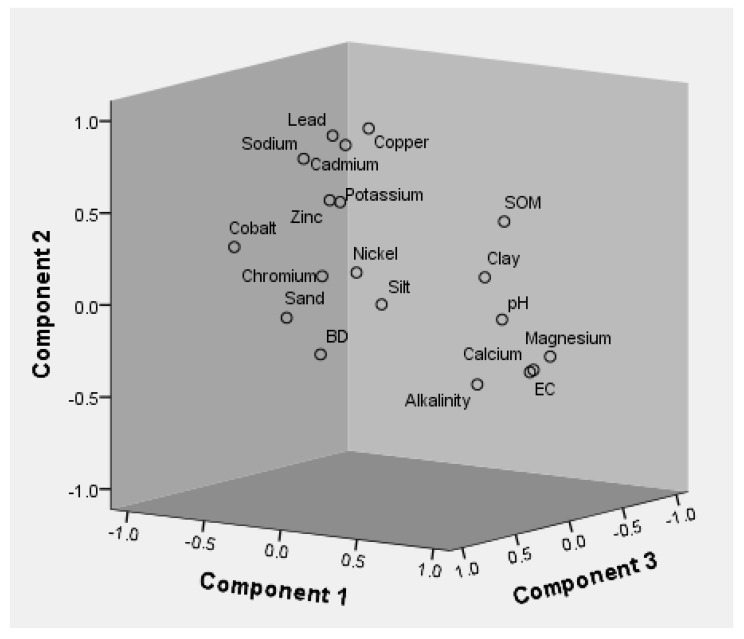
Loading plot of soil (Sampling 1) physico-chemical properties and heavy metals for principal component/factor analysis.

**Figure 7:**
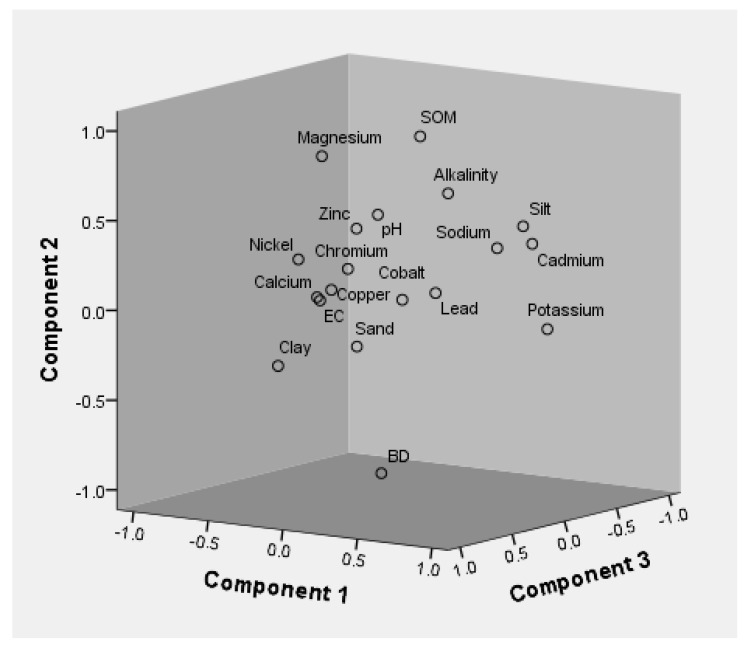
Loading plot of soil (Sampling 2) physico-chemical properties and heavy metals for principal component/factor analysis.

The variance in the soil quality parameters collected from the 8 different roadside sites during 2 sampling periods, viz., September 2018 (Sampling 1) and April 2019 (Sampling 2), was estimated following a factor analysis using principal component analysis as an extraction method and varimax as the rotation method (Figure 6 and Figure 7, and Table 10). The varifactor with an eigenvalue of more than 1 was taken into consideration, and factor loading was designated into various classes as weak (0.4–0.5), moderate (0.5–0.75) and strong (>0.75), as described by Liu et al. [93]. Factor analysis conducted on the parameters produced 4 and 5 factors explaining 94.88% and 92.98% of the cumulative variance, with an eigenvalue of more than 1 during Sampling 1 and 2, respectively. Factor 1 provided, during Sampling 1, 31.77% of the total variance with strong positive factor loadings of EC, SOM, calcium, and magnesium; strong negative loading of sand; moderate positive loadings of clay and alkalinity; moderate negative loading of Co; and weak negative loading of potassium; and, during Sampling 2, 21.88% of total variance with strong positive loadings of silt and potassium; strong negative loadings of clay and Ni; and moderate negative loading of Zn. During Sampling 1, factors 2, 3 and 4 explained 25.36%, 21.55% and 16.20% of the total variance, with strong positive factor loadings of Na, Cd, Cu, Pb; Cr, Ni and clay content, respectively (Table 10). Similarly, in the case of Sampling 2, factors 2, 3, 4 and 5 provided 21.64%, 18.10%, 16.15% and 15.21% of the total variance, with strong positive factor loadings of soil organic matter (SOM), magnesium; calcium, Cu; sand content, Co and Cr, Pb, respectively (Table 10). While studying the soils in the Northern Plateau of Spain, Santos-Frances et al. [94] discovered Co loading on factor/Principal component 1 and concluded that the source is primarily parent rock. Singh et al. [95], in their study on soils in the Varanasi area, demonstrated Cu and Pb loadings on factor 2 and Cr loadings on factor 4, and discovered that factor 2 is controlled by automobile emissions, while factor 4 is represented by parent material in addition to anthropogenic actions. The weathering of crash barriers and the abrasion of automobiles also contributed to higher levels of lead and zinc in the roadside soil [96,97]. Many researchers around the world reported traffic-related activities as a major source of Pb and Zn [34,75,98]. Similarly, various researchers throughout the world also reported factor analysis in the different sampling regions [32,79,99,100].

**Figure 8:**
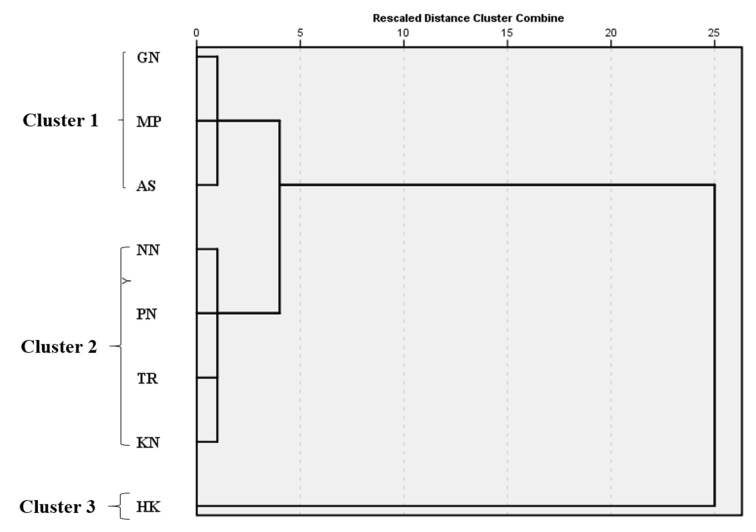
Dendrogram of cluster analysis showing the clustering of different roadside sampling sites during Sampling 1. Sample codes as mentioned in Table 1.

**Figure 9:**
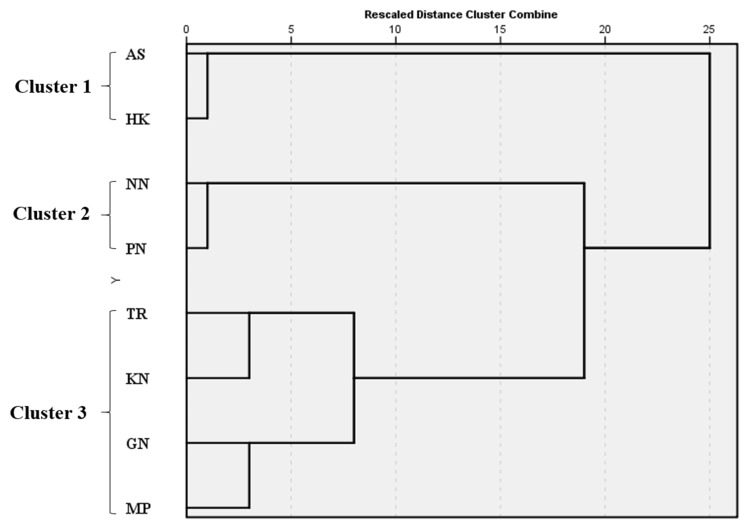
Dendrogram of cluster analysis showing the clustering of different roadside sampling sites during Sampling 2. Sample codes as mentioned in Table 1.

The hierarchical cluster analysis was used for the grouping of different sampling sites on the basis of soil quality parameters to identify the spatial variability. The current study categorized 8 roadside sites into statistically significant clusters based on their similarity in status, features and pollution source. During Sampling 1 (September 2018), 3 clusters were formed between 8 roadside sites; cluster 1 was formed by aggregating highly polluted sites, such as Geeta Nagar (GN), Madho Puri (MP) and Arvindra Street (AS); cluster 2 was formed by aggregating the extremely polluted sites, such as Nanak Nagar (NN), Pritam Nagar (PN), Tajpur Road (TR) and Kitchlu Nagar (KN); and cluster 3 contained only one site, Haibowal Kalan (HK), of the study area (Figure 8). Similarly, during Sampling 2 (April 2019), three clusters were constructed based upon their characteristics and pollution status (Figure 9). Two sites, Arvindra Street (AS) and Haibowal Kalan (HK), constituted cluster 1. Cluster 2 was formed by Nanak Nagar (NN) and Pritam Nagar (PN). Cluster 4 was the aggregation of highly polluted sites Tajpur Road (TR), Kitchlu Nagar (KN), Geeta Nagar (GN) and Madho Puri (MP). Similarly, cluster analysis was reported by various authors worldwide [77,101,102].

## 4. Conclusions

The roadside soil alongside the Buddha Nullah, Ludhiana was contaminated as a result of poor solid/liquid waste disposal and industrial activities. The high concentration of metals, such as Cd, Co, Cu, Pb and Zn, implies that the soil of the studied area was polluted with heavy metals, which arise from industrial activities and may have a direct influence on human health, groundwater, terrestrial and therefore ecological systems. This study warns that precautions must be taken to prevent soil pollution in the area. Igeo, CF, Cdeg, mCdeg, PI, PLI, ER_i_ and RI indices show that the soil in the studied area is moderate to extremely contaminated with few metals. The Igeo values of Cd, Cr, Co, Pb and Zn indicate no contamination to moderate contamination, whereas Cu and Ni shows moderate to heavy contamination in the studied area. The contamination factor values reveal that Cu and Ni show very high contamination in the study area. The results of the contamination degree and modified contamination degree show a considerable and moderate degree of contamination, respectively. On the basis of the results inferred from the Nemerow pollution index (PI), the soil samples under the study area were found to show moderate to heavy pollution. The results of ER_i_ and RI indicated a low to moderate risk of heavy metals in the studied soil samples. The Pearson correlation analysis shows that pH is correlated with Mg^2+^, Cr, Co, Ni and Zn, while EC shows a relationship with the sand content, alkalinity, Ca^2+^ and Mg^2+^. The clay content was observed to be statistically negatively correlated with the silt and sand contents in the roadside soil samples. Heavy metals were observed to be significantly correlated with each other, highlighting their similar origin.

## Figures and Tables

**Table 1:** Description of the sample codes and geographical location of the site of the sample collection.

S. No.	Name of Sampling Site	Sample Code	Location		Contributing Sources of Pollution
			Latitude	Longitude	
1	Site 1: Geeta Nagar	GN	30°55′08.5″ N	75°53′56.3″ E	Domestic and industrial
2	Site 2: Tajpur Road	TR	30°55′04.6″ N	75°53′08.9″ E	Dairy, domestic and industrial
3	Site 3: Madho Puri	MP	30°55′09.2″ N	75°51′59.4″ E	Domestic and industrial
4	Site 4: Arvindra Street	AS	30°55′48.5″ N	75°51′20.0″ E	Domestic and industrial
5	Site 5: Nanak Nagar	NN	30°55′42.2″ N	75°50′40.3″ E	Domestic and industrial
6	Site 6: Pritam Nagar	PN	30°55′16.4″ N	75°49′49.5″ E	Domestic and industrial
7	Site 7: Haibowal Kalan	HK	30°55′03.7″ N	75°48′24.4″ E	Dairy, domestic and industrial
8	Site 8: Kitchlu Nagar	KN	30°55′31.2″ N	75°47′31.5″ E	Dairy, domestic and industrial

**Table 2:** Descriptions of the soil contamination indices used in the study.

Indices	Formula	Description	Limit Values	Classification	References
Index of geoaccumulation (Igeo)	$\mathrm{Igeo}=log_{2}\;\left(\frac{\;C_{n}}{1.5\;B_{n}}\right)$ Where *C*_n_: content of heavy metal in soil; *B*_n_: background value; 1.5: constant	Igeo is a comparative analysis of the content of any heavy metal analyzed in an existing sample to pre-industrial.	<0	Class 0: practically uncontaminated	[47]
			0–1	Class 1: uncontaminated to moderately contaminated	
			1–2	Class 2: moderately contaminated	
			2–3	Class 3: moderately to heavily contaminated	
			3–4	Class 4: heavily contaminated	
			4–5	Class 5: heavily to extremely contaminated	
			>5	Class 6: extremely contaminated	
Contamination factor (CF)	$\mathrm{CF}=\frac{C_{i}}{C_{n}\;}$ Where *C_i_*: content of heavy metal in soil; *C_n_*: background value of heavy metal element *i*	CF provides the information to assess the pollution level of individual elements in the polluted soil as compared to the non-polluted soil.	<1	Class 1: low contamination	[48]
			1–3	Class 2: moderate contamination	
			3–6	Class 3: considerable contamination	
			>6	Class 4: very high contamination	
Contamination degree (Cdeg)	$C_{deg}=\sum_{i=1}^{n}CF_{i}$ Where *CF*: contamination factor of single heavy metal; *n*: number of heavy metals	Cdeg is the sum of all the contamination factors for a given set of soil samples.	<8	Low degree of contamination	[49,50]
			8–16	Moderate degree of contamination	
			16–32	Considerable degree of contamination	
			>32	Very high degree of contamination	
Modified contamination degree (mCdeg)	$mCd_{deg}=\frac{\sum_{i=1}^{i=n}CF_{i}}{n}$ Where *CF*: contamination factor of single heavy metal; *n*: number of heavy metals	It is the sum of all the contamination factors for a given set of samples divided by the number of analyzed metals.	<1.5	Nil to very low degree of contamination	[51]
			1.5–2	Low degree of contamination	
			2–4	Moderate degree of contamination	
			4–8	High degree of contamination	
			8 < 16	Very high degree of contamination	
			16–32	Extremely high degree of contamination	
			>32	Ultra high degree of contamination	
Nemerow’s pollution index (PI)	$PI=\sqrt{\frac{{\left(CFaver\right)}^{2}+{\left(CFmax\right)}^{2}}{2}}$ Where *CF_aver_*: average value of the contamination factor; *CF_max_*: maximum value of the contamination factor	It is the quantitative evaluation of the degree of pollution or contamination.	<0.7	Unpolluted	[50,52]
			0.7–1	Slightly unpolluted	
			1–2	Moderately polluted	
			2–3	Severely polluted	
			>3	Heavily polluted	
Pollution load index (PLI)	$PLI=\sqrt[{n}]{CF1\times CF2\times \ldots \times CFn}$ Where *CF*: contamination factor; *n*: number of metals	PLI calculates the level of metal contamination in the soil relatively based on all metals studied in the area.	<1	No pollution	[53]
			1–2	Moderate pollution	
			2–3	Heavy pollution	
			>3	Extremely heavy pollution	
Potential ecological risk factor (ER_i_)	$ER_{i}=CF_{i}\times Tr^{i}$ Where *Tr^i^*: toxicity response coefficient of heavy metal; *CF_i_*: contamination factor of heavy metal	ER_i_ is now widely used to assess the ecological risk of heavy metals in soil.	<40	Low potential ecological risk	[48]
			40–80	Moderate potential ecological risk	
			80–160	Considerable potential ecological risk	
			160–320	High potential ecological risk	
			>320	Very high potential ecological risk	
Potential ecological risk index (RI)	$RI=\sum_{i=1}^{n}ER_{i}$ Where *Er_i_*: potential ecological risk factor for heavy metal; *n*–number of analyzed heavy metals	RI initially is widely adopted to evaluate the potential ecological risk of the studied heavy metals in the soil.	<150	Low ecological risk	[48]
			15–300	Moderate ecological risk	
			300–600	Considerate ecological risk	
			>600	Very high ecological risk	

**Table 3:** Physico-chemical characteristics of roadside soil samples collected from the vicinity of Buddha Nullah Ludhiana, Punjab (India).

Site	pH	EC (mS/cm)	BD (g/cc)	Sand (%)	Silt (%)	Clay (%)	SOM (%)	T-A (mg/kg)	Ca^2+^ (mg/kg)	Mg^2+^ (mg/kg)	Na (mg/kg)	K (mg/kg)
Sampling 1												
GN	6.66 ± 0.01	0.49 ± 0.01	1.13 ± 0.00	16.97 ± 0.60	15.47 ± 0.71	67.56 ± 0.27	4.79 ± 0.41	2666.67 ± 166.67	293.92 ± 13.36	32.49 ± 8.12	584.83 ± 2.04	1242.17 ± 2.96
TR	7.03 ± 0.01	0.27 ± 0.00	0.97 ± 0.00	14.35 ± 0.72	17.26 ± 1.37	68.39 ± 0.70	8.65 ± 0.35	500.00 ± 0.00	173.68 ± 13.36	89.34 ± 21.49	548.17 ± 2.25	2507.25 ± 33.23
MP	7.22 ± 0.00	0.16 ± 0.00	1.27 ± 0.01	14.99 ± 0.30	8.58 ± 0.27	76.43 ± 0.41	1.99 ± 0.11	2166.67 ± 166.67	120.24 ± 0.00	32.49 ± 8.12	288.92 ± 0.68	1369.75 ± 14.89
AS	7.28 ± 0.01	0.37 ± 0.01	1.28 ± 0.01	23.49 ± 0.48	10.41 ± 1.90	66.10 ± 2.01	1.33 ± 0.06	1500.00 ± 0.00	146.96 ± 13.36	73.09 ± 14.07	233.92 ± 1.37	760.83 ± 2.21
NN	7.01 ± 0.01	0.18 ± 0.00	1.04 ± 0.00	25.75 ± 0.36	28.68 ± 0.65	45.58 ± 1.00	3.66 ± 0.18	1166.67 ± 166.67	133.60 ± 13.36	73.09 ± 14.07	289.67 ± 0.17	2560.75 ± 9.36
PN	6.89 ± 0.01	0.14 ± 0.00	1.17 ± 0.00	27.24 ± 0.88	16.43 ± 1.02	56.32 ± 1.41	5.89 ± 0.08	1666.67 ± 166.67	561.12 ± 23.14	89.34 ± 8.12	338.75 ± 3.44	2800.83 ± 11.69
HK	7.08 ± 0.00	2.60 ± 0.00	1.01 ± 0.00	1.66 ± 0.58	13.75 ± 0.59	84.59 ± 1.17	9.74 ± 0.10	5000.00 ± 0.00	3139.60 ± 66.80	609.12 ± 70.33	169.75 ± 1.89	249.83 ± 1.69
KN	7.06 ± 0.01	0.66 ± 0.00	1.08 ± 0.00	29.58 ± 0.29	22.31 ± 0.02	48.11 ± 0.27	1.79 ± 0.12	833.33 ± 166.67	574.48 ± 13.36	89.34 ± 8.12	209.00 ± 4.02	1758.50 ± 4.59
Sampling 2												
GN	7.32 ± 0.01	0.65 ± 0.00	1.12 ± 0.00	16.17 ± 0.89	15.92 ± 0.98	67.91 ± 1.04	3.96 ± 0.31	2500.00 ± 288.68	240.48 ± 0.00	219.28 ± 0.00	518.58 ± 4.92	1168.00 ± 3.92
TR	7.38 ± 0.01	0.63 ± 0.00	0.90 ± 0.01	3.50 ± 0.09	60.43 ± 0.65	36.07 ± 0.74	10.73 ± 0.16	3333.33 ± 166.67	293.92 ± 13.36	235.53 ± 8.12	507.08 ± 2.07	2360.47 ± 18.19
MP	7.78 ± 0.01	1.77 ± 0.01	0.89 ± 0.00	9.63 ± 0.15	38.37 ± 0.21	52.00 ± 0.28	9.33 ± 0.09	3000.00 ± 0.00	935.20 ± 13.36	422.32 ± 8.12	255.62 ± 2.73	1207.32 ± 5.72
AS	7.45 ± 0.01	2.16 ± 0.01	1.23 ± 0.01	3.61 ± 0.27	29.09 ± 1.17	67.31 ± 0.99	4.64 ± 0.14	1916.67 ± 83.33	360.72 ± 0.00	170.55 ± 0.00	216.08 ± 3.25	699.18 ± 6.85
NN	7.18 ± 0.01	0.94 ± 0.00	1.22 ± 0.00	12.73 ± 1.51	32.10 ± 0.36	55.17 ± 1.31	2.46 ± 0.17	1583.33 ± 83.33	774.88 ± 13.36	97.46 ± 0.00	259.63 ± 1.87	2423.47 ± 9.34
PN	7.50 ± 0.06	0.21 ± 0.00	1.13 ± 0.00	7.46 ± 0.87	39.05 ± 3.09	53.50 ± 2.63	3.31 ± 0.40	2083.33 ± 83.33	227.12 ± 26.72	178.67 ± 16.24	312.13 ± 3.22	2637.22 ± 7.93
HK	7.13 ± 0.01	0.16 ± 0.07	1.11 ± 0.07	9.97 ± 0.61	10.84 ± 0.56	79.20 ± 0.99	4.38 ± 0.35	1416.67 ± 83.33	280.56 ± 0.00	227.40 ± 8.12	151.82 ± 2.54	219.03 ± 3.84
KN	7.34 ± 0.01	0.36 ± 0.00	1.08 ± 0.00	22.12 ± 1.33	29.68 ± 2.41	48.20 ± 2.38	4.38 ± 0.23	3666.67 ± 166.67	334.00 ± 13.36	146.19 ± 0.00	182.57 ± 2.15	1577.07 ± 14.33
Limits	6.5–8.5 ^a^	4.5 ^b^	-	-	-	-	-	-	0–3500 ^c^	0–500 ^c^	0–300 ^c^	0–450 ^c^

(EC: electrical conductivity’ BD: bulk density, SOM: soil organic matter, T-A: alkalinity, Ca^2+^: calcium, Mg^2+^: magnesium, Na: sodium and K: potassium); ^a^ Ramachandra et al., 2012 [54] (optimum range); ^b^ Brouwer et al., 1985 [55] (salt concentration limit for the soil extract of non-saline soils); ^c^ Alghobar and Suresha 2017 [62] (as per ISI, 1983 [60] and Awashthi, 2000 [61]) (Indian standards). Sample codes as mentioned in Table 1.

**Table 4:** Heavy metal contents (mg/kg) of roadside soil samples collected from the vicinity of Buddha Nullah Ludhiana, Punjab (India).

Site	Cadmium	Chromium	Cobalt	Copper	Lead	Nickel	Zinc
Sampling 1							
GN	0.31 ± 0.01	104.62 ± 0.25	11.29 ± 0.17	44.04 ± 0.28	57.25 ± 0.76	46.73 ± 0.16	246.84 ± 0.15
TR	0.46 ± 0.02	84.69 ± 0.39	8.24 ± 0.44	67.50 ± 0.46	69.17 ± 1.12	26.99 ± 0.12	196.97 ± 0.18
MP	0.08 ± 0.01	77.18 ± 0.61	8.03 ± 0.30	16.85 ± 0.58	34.75 ± 4.27	13.01 ± 0.35	73.23 ± 0.21
AS	0.14 ± 0.01	73.53 ± 0.35	6.15 ± 0.14	10.05 ± 0.23	19.42 ± 0.79	9.18 ± 0.11	59.42 ± 0.46
NN	0.11 ± 0.01	79.48 ± 0.42	8.61 ± 0.12	20.82 ± 0.21	32.33 ± 1.17	18.51 ± 0.21	73.64 ± 0.61
PN	0.36 ± 0.02	83.77 ± 0.49	10.36 ± 0.26	24.86 ± 0.09	31.25 ± 1.01	21.21 ± 0.10	89.23 ± 1.39
HK	0.07 ± 0.01	86.46 ± 0.64	4.15 ± 0.12	13.75 ± 0.22	14.75 ± 1.23	31.03 ± 0.71	99.69 ± 0.75
KN	0.13 ± 0.01	81.44 ± 0.56	8.51 ± 0.07	15.21 ± 0.05	19.42 ± 0.88	21.23 ± 0.17	107.74 ± 0.37
Sampling 2							
GN	0.22 ± 0.01	93.85 ± 0.18	6.83 ± 0.09	79.37 ± 0.09	47.75 ± 0.95	134.08 ± 0.82	303.58 ± 0.28
TR	0.29 ± 0.01	30.58 ± 0.71	6.87 ± 0.07	77.14 ± 0.08	36.67 ± 0.88	33.44 ± 0.09	189.02 ± 0.12
MP	0.08 ± 0.00	76.91 ± 0.08	7.63 ± 0.07	198.29 ± 0.36	43.75 ± 0.66	87.74 ± 0.13	134.61 ± 0.18
AS	0.09 ± 0.01	28.83 ± 0.51	4.92 ± 0.08	87.68 ± 0.10	27.42 ± 1.30	18.97 ± 0.12	61.69 ± 0.11
NN	0.04 ± 0.01	35.83 ± 0.71	7.84 ± 0.09	151.79 ± 0.51	38.42 ± 0.68	32.83 ± 0.09	58.21 ± 1.82
PN	0.26 ± 0.02	24.62 ± 0.08	8.37 ± 0.05	77.12 ± 0.06	33.25 ± 0.76	29.06 ± 0.07	53.88 ± 0.22
HK	0.03 ± 0.00	9.32 ± 0.08	8.87 ± 0.07	52.07 ± 0.09	8.83 ± 0.58	182.88 ± 0.15	287.30 ± 0.28
KN	0.03 ± 0.01	58.67 ± 0.65	11.43 ± 0.08	85.58 ± 0.08	41.75 ± 0.87	120.42 ± 0.13	232.73 ± 0.12
Agarwal (2009)	0.06	100	8	20	10	-	50
Indian-Awasthi (2000)	3–6	-	-	135–270	250–500	-	300–600
European Union (2009)	1.0	100	50	100	100	-	300
Swedish Limits	0.4	120	30	100	80	-	350

Agarwal, 2009 [71] (typical soil concentration); Indian-Awasthi 2000 [61]; European Union 2009 [83]; SGV-Swedish limits https://www.naturvardsverket.se/Documents/publikationer/620-5053-2.pdf (accessed on 24 July 2021) Bhagure and Mirgane, 2011, [84]. Sample codes as mentioned in Table 1.

**Table 5:** Index of the geoaccumulation (Igeo) of roadside soil samples collected from the vicinity of Buddha Nullah stream, Ludhiana (Punjab), India, during Sampling 1 (S1: September 2018) and Sampling 2 (S2: April 2019).

Sample Code	Cd		Cr		Co		Cu		Pb		Ni		Zn	
	S1	S2	S1	S2	S1	S2	S1	S2	S1	S2	S1	S2	S1	S2
GN	1.07	0.56	0.99	0.84	−0.41	−1.14	0.23	1.08	0.93	0.67	0.19	2.16	0.37	0.45
TR	1.64	0.99	0.69	−0.78	−0.86	−1.13	0.85	1.04	1.21	0.29	−0.05	0.16	0.27	0.25
MP	−0.82	−0.97	0.56	0.55	−0.90	−0.97	−1.15	2.40	0.21	0.54	−0.36	1.55	−0.16	0.10
AS	−0.05	−0.68	0.49	−0.86	−1.29	−1.61	−1.90	1.23	−0.63	−0.13	−0.51	−0.66	−0.25	−0.24
NN	−0.44	−1.82	0.60	−0.55	−0.80	−0.94	−0.85	2.02	0.11	0.36	−0.21	0.13	−0.16	−0.26
PN	1.29	0.81	0.67	−1.09	−0.53	−0.84	−0.59	1.04	0.06	0.15	−0.15	−0.05	−0.08	−0.30
HK	−1.14	−2.56	0.72	−2.49	−1.85	−0.76	−1.45	0.47	−1.02	−1.76	0.01	2.61	−0.03	0.43
KN	−0.14	−2.14	0.63	0.16	−0.82	−0.39	−1.30	1.19	−0.63	0.48	−0.15	2.01	0.01	0.34

Sample codes as mentioned in Table 1.

**Table 6:** Contamination factor (CF), of roadside soil samples collected from the vicinity of Buddha Nullah stream, Ludhiana (Punjab), India, during Sampling 1 (S1: September 2018) and Sampling 2 (S2: April 2019).

Sample Code	Cd		Cr		Co		Cu		Pb		Ni		Zn	
	S1	S2	S1	S2	S1	S2	S1	S2	S1	S2	S1	S2	S1	S2
GN	3.15	2.21	2.99	2.68	1.13	0.68	1.76	3.17	2.86	2.39	2.34	6.70	3.48	4.28
TR	4.68	2.98	2.42	0.87	0.82	0.69	2.70	3.09	3.46	1.83	1.35	1.67	2.77	2.66
MP	0.85	0.77	2.21	2.20	0.80	0.76	0.67	7.93	1.74	2.19	0.65	4.39	1.03	1.90
AS	1.45	0.94	2.10	0.82	0.62	0.49	0.40	3.51	0.97	1.37	0.46	0.95	0.84	0.87
NN	1.11	0.43	2.27	1.02	0.86	0.78	0.83	6.07	1.62	1.92	0.93	1.64	1.04	0.82
PN	3.66	2.64	2.39	0.70	1.04	0.84	0.99	3.08	1.56	1.66	1.06	1.45	1.26	0.76
HK	0.68	0.26	2.47	0.27	0.42	0.89	0.55	2.08	0.74	0.44	1.55	9.14	1.40	4.05
KN	1.36	0.34	2.33	1.68	0.85	1.14	0.61	3.42	0.97	2.09	1.06	6.02	1.52	3.28

Sample codes as mentioned in Table 1.

**Table 7:** Potential ecological risk factor (ERi) and potential ecological risk index (RI) of roadside soil samples collected from the vicinity of Buddha Nullah stream, Ludhiana (Punjab), India, during Sampling 1 (S1: September 2018) and Sampling 2 (S2: April 2019).

Sample Code	Potential Ecological Risk Factor (ERi)														RI	
	Cd		Cr		Co		Cu		Pb		Ni		Zn			
	S1	S2	S1	S2	S1	S2	S1	S2	S1	S2	S1	S2	S1	S2	S1	S2
GN	94.39	66.33	5.98	5.36	5.65	3.41	8.81	15.87	14.31	11.94	11.68	33.52	3.48	4.28	144.29	140.71
TR	140.31	89.29	4.84	1.75	4.12	3.43	13.50	15.43	17.29	9.17	6.75	8.36	2.77	2.66	189.58	130.09
MP	25.51	22.96	4.41	4.39	4.01	3.82	3.37	39.66	8.69	10.94	3.25	21.94	1.03	1.90	50.27	105.60
AS	43.37	28.06	4.20	1.65	3.08	2.46	2.01	17.54	4.85	6.85	2.29	4.74	0.84	0.87	60.64	62.17
NN	33.16	12.76	4.54	2.05	4.30	3.92	4.16	30.36	8.08	9.60	4.63	8.21	1.04	0.82	59.92	67.72
PN	109.69	79.08	4.79	1.41	5.18	4.18	4.97	15.42	7.81	8.31	5.30	7.26	1.26	0.76	139.00	116.43
HK	20.41	7.65	4.94	0.53	2.08	4.43	2.75	10.41	3.69	2.21	7.76	45.72	1.40	4.05	43.02	75.01
KN	40.82	10.20	4.65	3.35	4.25	5.72	3.04	17.12	4.85	10.44	5.31	30.10	1.52	3.28	64.44	80.21

Sample codes as mentioned in Table 1.

**Table 8:** The Pearson correlation coefficient among the roadside soil quality parameters collected from the vicinity of Buddha Nullah, Ludhiana (Punjab), India, sampled during September 2018 (Sampling 1).

	pH	EC	Sand	Silt	Clay	BD	SOM	T-A	Ca^2+^	Mg^2+^	Na	K	Cd	Cr	Co	Cu	Pb	Ni	Zn
pH	1																		
EC	0.67	1																	
Sand	−0.06	−0.72 *	1																
Silt	−0.33	−0.15	0.47	1															
Clay	0.20	0.56	−0.91 **	−0.80 *	1														
BD	0.34	−0.40	0.29	−0.61	0.10	1													
SOM	−0.32	0.57	−0.68	0.01	0.46	−0.71 *	1												
T-A	−0.09	0.84 **	−0.77 *	−0.39	0.71 *	−0.05	0.46	1											
Ca^2+^	0.04	0.98 **	−0.69	−0.14	0.54	−0.39	0.63	0.85 **	1										
Mg^2+^	0.13	0.97 **	−0.72 *	−0.12	0.55	−0.43	0.66	0.81 *	0.99 **	1									
Na	−0.67	−0.42	−0.05	0.01	0.03	−0.21	0.27	−0.28	−0.45	−0.46	1								
K	−0.30	−0.68	0.60	0.58	−0.69	−0.25	0.01	−0.73 *	−0.57	−0.56	0.37	1							
Cd	−0.54	−0.39	0.10	0.0	−0.09	−0.28	0.43	−0.40	−0.34	−0.34	0.82 *	0.57	1						
Cr	−0.90 **	0.18	−0.28	0.05	0.17	−0.32	0.40	0.33	0.15	0.06	0.67	−0.08	0.43	1					
Co	−0.73 *	−0.70	0.55	0.28	−0.51	0.10	−0.23	−0.48	−0.65	−0.74 *	0.67	0.64	0.57	0.50	1				
Cu	−0.50	−0.27	−0.17	0.10	0.07	−0.50	0.5	−0.32	−0.29	−0.26	0.90 **	0.44	0.86 **	0.51	0.44	1			
Pb	−0.51	−0.44	−0.11	0.04	0.06	−0.29	0.31	−0.36	−0.46	−0.44	0.96 **	0.44	0.79 *	0.52	0.57	0.95 **	1		
Ni	−0.85 **	0.33	−0.39	0.10	0.22	−0.49	0.54	0.39	0.29	0.22	0.61	−0.12	0.40	0.98 **	0.35	0.53	0.49	1	
Zn	−0.74 *	−0.03	−0.24	0.03	0.15	−0.41	0.40	−0.01	−0.10	−0.14	0.88 **	0.07	0.66	0.87 **	0.49	0.82 *	0.80 *	0.86 **	1

(EC: electrical conductivity; BD: bulk density; SOM: soil organic matter; T-A: alkalinity; Ca^2+^: calcium; Mg^2+^: magnesium; Na: sodium; K: potassium; Cd: cadmium; Cr: chromium; Co: cobalt; Cu: copper; Pb: lead; Ni: nickel and Zn: zinc). * Correlation is significant at the 0.05 level (2-tailed). ** Correlation is significant at the 0.01 level (2-tailed).

**Table 9:** Pearson correlation coefficient among the roadside soil quality parameters collected from the vicinity of Buddha Nullah, Ludhiana (Punjab), India, sampled during April 2019 (Sampling 2).

	pH	EC	Sand	Silt	Clay	BD	SOM	T-A	Ca^2+^	Mg^2+^	Na	K	Cd	Cr	Co	Cu	Pb	Ni	Zn
pH	1																		
EC	0.55	1																	
Sand	−0.26	−0.39	1																
Silt	0.44	0.13	−0.47	1															
Clay	−0.38	0.03	0.06	−0.91 **	1														
BD	−0.54	0.02	0.13	−0.56	0.57	1													
SOM	0.53	0.26	−0.42	0.67	−0.56	−0.91 **	1												
T-A	0.44	−0.03	0.31	0.52	−0.73 *	−0.69	0.60	1											
Ca^2+^	0.41	0.54	0.02	0.16	−0.19	−0.23	0.22	−0.00	1										
Mg^2+^	0.72 *	0.33	−0.22	0.16	−0.07	−0.787 *	0.71 *	0.31	0.42	1									
Na	0.07	−0.13	−0.16	0.39	−0.37	−0.34	0.36	0.31	−0.26	0.11	1								
K	0.09	−0.28	−0.05	0.70	−0.77 *	−0.11	0.04	0.24	0.08	−0.30	0.40	1							
Cd	0.22	−0.22	−0.42	0.53	−0.41	−0.32	0.33	0.24	−0.46	0.09	0.85 **	0.51	1						
Cr	0.40	0.21	0.53	−0.12	−0.12	−0.28	0.14	0.51	0.28	0.39	0.40	−0.06	0.07	1					
Co	−0.21	−0.66	0.73 *	−0.15	−0.17	−0.12	−0.21	0.33	−0.05	−0.15	−0.40	0.13	−0.36	0.02	1				
Cu	0.57	0.57	0.04	0.24	−0.29	−0.29	0.26	0.13	0.97 **	0.47	−0.13	0.17	−0.31	0.43	−0.09	1			
Pb	0.44	0.18	0.39	0.34	−0.57	−0.27	0.17	0.62	0.31	0.13	0.53	0.48	0.29	0.82 *	0.01	0.49	1		
Ni	−0.33	−0.43	0.56	−0.71 *	0.54	−0.11	−0.12	0.00	−0.18	0.23	−0.10	−0.65	−0.37	0.24	0.49	−0.26	−0.26	1	
Zn	−0.36	−0.48	0.48	−0.45	0.29	−0.27	0.11	0.26	−0.40	0.18	0.22	−0.49	−0.02	0.33	0.34	−0.43	−0.07	0.87 **	1

(EC: electrical conductivity; BD: bulk density; SOM: soil organic matter; T-A: alkalinity; Ca^2+^: calcium; Mg^2+^: magnesium; Na: sodium; K: potassium; Cd: cadmium; Cr: chromium; Co: cobalt; Cu: copper; Pb: lead; Ni: nickel and Zn: zinc). * Correlation is significant at the 0.05 level (2-tailed). ** Correlation is significant at the 0.01 level (2-tailed).

**Table 10:** Factor analysis of the complete data set of roadside soil quality parameters alongside Buddha Nullah, Ludhiana (Punjab), India, sampled in September 2018 (Sampling 1) and April 2019 (Sampling 2).

Parameter	Sampling 1				Sampling 2				
	Factor 1	Factor 2	Factor 3	Factor 4	Factor 1	Factor 2	Factor 3	Factor 4	Factor 5
pH	0.04	−0.26	−0.91 ***	0.26	0.19	0.57 **	0.47 *	−0.29	0.25
EC	0.93 ***	−0.30	0.10	0.14	−0.06	0.10	0.67 **	−0.63 **	0.10
Sand	−0.78 ***	−0.21	−0.07	−0.52 **	−0.24	−0.26	0.07	0.78 ***	0.50 *
Silt	−0.03	−0.03	0.13	−0.96 ***	0.84 ***	0.51 **	0.01	−0.15	−0.04
Clay	0.55 **	0.16	−0.02	0.82 ***	−0.84 ***	−0.45 *	−0.04	−0.19	−0.19
Bulk density	−0.63 **	−0.40 **	−0.16	0.61 **	−0.12	−0.97 ***	−0.00	−0.12	−0.13
Soil organic matter	0.80 ***	0.51 **	0.16	−0.08	0.16	0.94 ***	0.03	−0.18	0.01
Alkalinity	0.72 **	−0.36	0.30	0.44 *	0.29	0.63 **	−0.05	0.37	0.45*
Calcium	0.93 ***	−0.30	0.08	0.09	0.08	0.16	0.90 ***	−0.01	0.11
Magnesium	0.96 ***	−0.24	−0.04	0.08	−0.30	0.82 ***	0.30	−0.19	0.13
Sodium	−0.26	0.80 ***	0.53 **	0.07	0.28	0.25	−0.55 **	−0.36	0.60 **
Potassium	−0.44 *	0.46 *	−0.07	−0.68 **	0.93 ***	−0.07	−0.09	0.11	0.17
Cadmium	−0.17	0.84 ***	0.26	−0.13	0.45 *	0.28	−0.64 **	−0.41	0.27
Chromium	0.16	0.26	0.95 ***	0.07	−0.20	0.19	0.20	0.07	0.94 ***
Cobalt	−0.67 **	0.28	0.59 **	−0.25	0.01	0.01	−0.01	0.98 ***	−0.05
Copper	−0.01	0.95 ***	0.28	−0.08	0.16	0.21	0.88 ***	−0.06	0.28
Lead	−0.21	0.90 ***	0.33	0.03	0.38	0.12	0.20	0.06	0.89 ***
Nickel	0.34	0.29	0.89 ***	0.01	−0.82 ***	0.13	−0.20	0.51 **	0.06
Zinc	0.05	0.63 **	0.72 **	0.07	−0.64 **	0.27	−0.51 **	0.39	0.25
Eigenvalue	6.04	4.82	4.10	3.08	4.16	4.11	3.44	3.07	2.89
% Total Variance	31.77	25.36	21.55	16.20	21.88	21.64	18.10	16.15	15.21
Cumulative % Variance	31.77	57.13	78.68	94.88	21.88	43.52	61.62	77.78	92.98

Extraction method: principal component analysis. Rotation method: Varimax with Kaiser normalization. *, **, *** represent weak, moderate and strong factor loadings, respectively.

## Data Availability

The data presented in this study are available on request from the corresponding author.
